# Digital financial literacy and household consumption: Total expenditure versus consumption structure in China

**DOI:** 10.1371/journal.pone.0357484

**Published:** 2026-09-01

**Authors:** Yan Shen, Wenxiu Hu, Xin Wang

**Affiliations:** School of Economics and Management, Xi’an University of Technology, Xi’an, China; Universiti Malaysia Sabah, MALAYSIA

## Abstract

Using data from the China Household Finance Survey, this study examines how digital financial literacy relates to household consumption. Digital financial literacy is positively associated with total consumption expenditure, especially among low-income households. Borrowing is the main pathway linking digital financial literacy to consumption, while investment and insurance play smaller roles. In contrast, the evidence for a systematic shift in the full consumption structure is not robust. The study suggests that digital financial literacy programmes can serve as an effective demand-side tool for household consumption, especially for low-income groups, but achieving structural changes in consumption likely requires complementary policies in education, health, and culture. Facilitating responsible digital credit access and building trust in digital insurance products are important for the link between digital financial literacy and higher household consumption.

## 1. Introduction

Rooted in the Permanent Income Hypothesis, consumption decisions are related to individuals’ long-term income expectations, which encompass both labor income and property income. However, as labor’s share of GDP has declined by 0.6 percentage points over the past decade, according to the *World Employment and Social Outlook: May 2025 Update* [1], and wage growth has stagnated, the potential role of property income in household consumption has received insufficient scholarly attention. This study investigates whether digital financial literacy (DFL) is associated with total consumption expenditure (CE) and consumption structure. The potential channel is financial behaviors that are related to property income. Thus, DFL provides a pathway beyond wage-driven growth.

Financial behaviors, such as borrowing, investment, and insurance, are associated with household consumption through various channels. For instance, borrowing alleviates short-term liquidity constraints [2,3], investment generates property income [4,5], and insurance reduces precautionary savings [6]. These pathways have been well documented in the literature. However, in the digital economy, effectively engaging in these behaviors often requires adequate DFL. Thus, understanding how DFL relates to these behaviors is key to explaining its association with household consumption.

Financial literacy is significantly related to greater participation in formal financial markets [7]. Traditional financial literacy metrics fail to address the complexities facing consumers in digital financial environments. It is essential to integrate digital and financial literacy [8]. The Alliance for Financial Inclusion (AFI) defines digital financial literacy as acquiring knowledge, skills, confidence, and competencies to safely use digitally delivered financial products and services, to make informed financial decisions [9]. Higher DFL levels are significantly associated with residents’ engagement in financial activities for economic benefit [10–12]. High DFL is associated with more rational decision-making in financial investment, while low DFL is correlated with herd investing [13]. Given that digital finance is associated with consumption, the current study aims to explore potential pathways through which DFL may be related to consumption by integrating digital financial literacy, financial behavior, and consumption into a unified analytical framework.

While studies have predominantly focused on the absolute level of consumption, this research characterizes consumption behavior from two complementary dimensions: total consumption expenditure and consumption structure. The former pertains to “how much is consumed” and represents a direct indicator of consumption capacity. The latter refers to the proportional allocation of expenditures across different consumption types, reflecting the internal composition and hierarchy of consumer needs; thus, consumption structure addresses the question of “what is consumed.” Some existing studies have examined the relationship between digital finance (or financial literacy) and consumption structure, finding positive relationships and identifying various transmission pathways [14–16]. However, they rarely take DFL as the core concept, nor do they rigorously distinguish between the links with total expenditure versus consumption structure. Moreover, most studies treat the population as homogeneous, neglecting heterogeneity across demographic groups. To fill these gaps, this study empirically examines the associations, pathways, and heterogeneities of DFL with household consumption using Chinese survey data.

This study makes four contributions. First, our results show a robust positive association between DFL and total consumption expenditure, but no robust evidence that DFL is associated with changes in consumption structure (i.e., the shares of subsistence, developmental, and enjoyment spending). This finding challenges the widely held view that digital finance automatically promotes consumption upgrading. Second, borrowing is the main pathway between DFL and consumption, while investment and insurance play smaller roles, consistent with a liquidity-constraint interpretation. Third, we document heterogeneous associations: the DFL–CE link is strongest for low-income households, while urban-rural differences are only marginally significant, and regional or educational differences are not significant. These patterns offer preliminary insights for targeted policy interventions. Thus, targeted DFL programs for disadvantaged groups may yield the highest returns. Fourth, this study is the first to distinguish total consumption expenditure from consumption structure in the DFL literature. It reveals that DFL is associated with a lower subsistence consumption proportion and a higher enjoyment consumption proportion, yet the evidence for a systematic shift in the full consumption structure is not robust.

## 2. Theoretical analysis and research hypotheses

### 2.1. Conceptual framework

We identify three potential channels through which DFL may be associated with household consumption.

Borrowing (liquidity constraints). Digital credit lowers transaction costs and reduces information frictions [17]. DFL is associated with better evaluation of credit terms and may help individuals avoid unfavorable lending conditions [18]. Relaxing liquidity constraints [2] is in turn associated with higher current consumption, especially for basic needs.

Investment (property income). DFL is associated with greater participation in risky asset markets [19], which may generate property income. Mental accounting suggests that such gains are often allocated to enjoyment consumption, such as travel or leisure [20].

Insurance (precautionary savings). DFL is associated with improved risk perception and insurance literacy [21]. This may allow households to reduce costly precautionary savings and replace them with lower-premium insurance [22], potentially freeing up income for current consumption.

**Fig 1:**
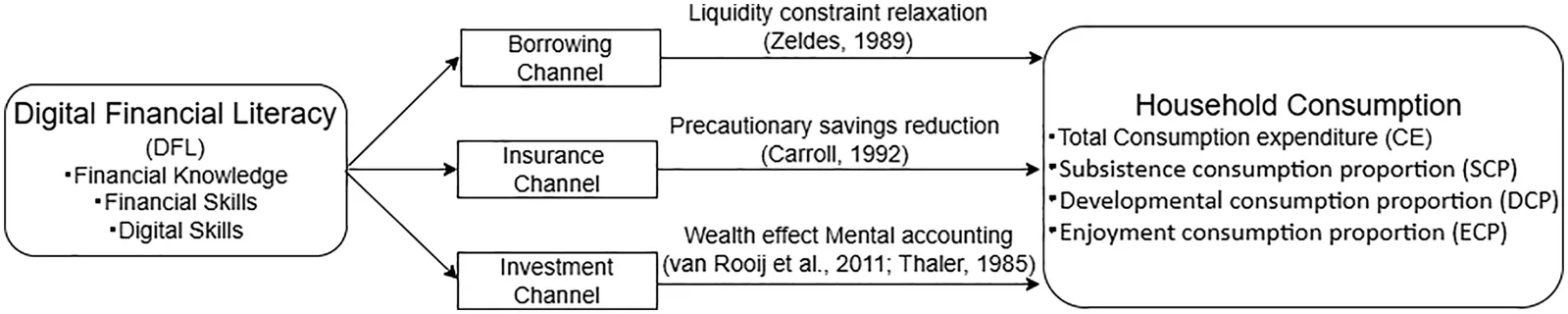
Conceptual framework of DFL’s association with household consumption.

Taken together, these channels suggest that DFL may be associated with household consumption outcomes, namely CE and the composition of spending across subsistence, developmental, and enjoyment categories. Fig 1 summarizes the framework.

### 2.2. Hypotheses development

Previous research using CHFS data shows that consumers’ digital financial capability is positively associated with total household consumption and online consumption [23]. The use of digital finance is associated with consumption upgrading and adjustments in household financial asset allocation, including participation in risk markets and greater portfolio diversity [16]. Higher DFL is related to consumers’ capacity to accurately assess the risk-return characteristics of credit instruments and wealth management products [24]. This allows them to allocate financial resources more efficiently while safeguarding capital security. This process releases consumption demand that had been suppressed by liquidity constraints. DFL is positively associated with current saving and spending behavior. It is also related to future savings and spending foresight [25]. Based on this evidence, we propose the following hypothesis:

When a country reaches the “stage of maturity,” consumption patterns are observed to shift toward durable goods and services [26]. Goods can be classified into necessities and non-necessities, which extends Maslow’s hierarchy of needs and links the income elasticity of different consumption types to need fulfillment at various hierarchical levels [27]. Based on these perspectives, Chinese scholars in the 1980s posited a tripartite classification of consumption—subsistence, developmental, and enjoyment—according to the level of needs satisfied [28]. The specific expenditure items are defined in the subsection titled “Dependent Variables.” In this framework, a fall in the subsistence consumption proportion (SCP) is associated with reduced life pressures; a rise in the developmental proportion (DCP) is associated with greater emphasis on self-improvement; and an increase in the enjoyment proportion (ECP) is associated with a transition from material to spiritual fulfillment.

**H1a:** Higher digital financial literacy is positively associated with household total consumption expenditure.

However, the literature on DFL and consumption structure is still nascent. Given that DFL is related to financial behavior and household resource allocation, it is reasonable to expect that higher DFL may also be associated with how households allocate spending across these three categories. For instance, through investment, borrowing, and insurance, DFL may be associated with increases in households’ disposable resources, which could in turn be associated with shifts in the allocation of spending across the three categories. For instance, it might be associated with a lower subsistence share and a higher enjoyment share, though the opposite patterns cannot be ruled out theoretically. Nevertheless, given the cross-sectional nature of our data, we treat these expected directions as tentative rather than confirmatory. Therefore, we state H1b as follows:

**H1b:** Higher DFL may be associated with changes in the consumption structure.

The three pathways illustrated in Fig 1 also point to possible associations between DFL and household consumption. Borrowing, investment, and insurance may each serve as a potential pathway. This yields the following hypotheses:

**H2a (Borrowing)**: Higher digital financial literacy is positively associated with borrowing, and borrowing is positively associated with total consumption expenditure and consumption structure.

**H2b (Investment)**: Higher digital financial literacy is positively associated with investment, and investment is positively associated with total consumption expenditure and consumption structure.

**H2c (Insurance)**: Higher digital financial literacy is positively associated with insurance, and insurance is positively associated with total consumption expenditure and consumption structure.

## 3. Data and Methodology

### 3.1. Data sources

This study uses data from the 2019 wave of the China Household Finance Survey (CHFS), a nationally representative survey conducted by Southwestern University of Finance and Economics. The CHFS collects detailed information on household assets, liabilities, income, expenditure, insurance, financial literacy, and demographic characteristics. We restrict the sample to respondents named as household heads. Observations with missing values on key variables, responses coded as “unknown” or “not applicable”, and implausible negative values are excluded. To mitigate the influence of outliers, household income and asset variables are winsorized at the 1st and 99th percentiles. The final sample comprises 14,956 households after excluding missing values and outliers. When all control variables are included, the sample size is 14,903 due to missing values in some control variables (mainly health status and family size).

### 3.2. Variable selection and statistical description

#### 3.2.1 Dependent variables.

The study focuses on two dependent variables: total consumption expenditure (CE) and consumption structure. CE is measured as the natural logarithm of total household expenditure across all consumption categories.

Based on the CHFS questionnaire content, consumption structure is captured by the proportion of three consumption types: subsistence, developmental, and enjoyment. Following the classification used in our hypothesis development, we define:

Subsistence consumption proportion (SCP) refers to the proportion of household expenditure allocated to basic necessities that satisfy fundamental physiological and safety needs. It includes spending on food, clothing, housing, and household equipment and services. These items are essential for daily survival and basic living standards.

Developmental consumption proportion (DCP) captures the proportion of expenditure aimed at improving human capital, long-term well-being, and future productivity. It consists of education, transportation, communication, and medical expenditures. (Medical expenditure is classified as developmental because, like education, it is associated with health capital and productivity; although a small portion may be involuntary, its primary function is human capital investment.)

The enjoyment consumption proportion (ECP) represents the proportion of expenditure devoted to leisure, recreation, and spiritual fulfillment. It includes entertainment, tourism, and voluntary wellness-related healthcare (e.g., preventive health checks, fitness activities, and spa services). These items go beyond basic needs and development and are associated with quality of life and subjective well-being.

#### 3.2.2 Explanatory Variable.

The explanatory variable is digital financial literacy (DFL). Drawing on the methodology devised by Lyons and Kass-Hanna (2021), DFL is measured across three dimensions: financial knowledge (comprehension of interest rates, inflation, and risk), financial skills (experience in stock/fund investment or using credit cards), and digital skills (proficiency in digital payment tools and online investment platforms). We acknowledge that the CHFS data do not contain direct measures of advanced competencies such as digital security or algorithmic literacy. Our DFL index therefore captures basic dimensions. This limitation is further discussed in the Limitations section.

To quantify DFL, factor analysis was conducted based on 12 measurement variables derived from the sample of 15,114 participants. The variables were grouped into three domains (see Table 1). Factor extraction was performed using the iterative principal component method, with varimax rotation then undertaken to enhance the interpretability of the factor structure. Given that FK1 (Focus on financial information) is an ordinal variable rated on a scale of 1–5, and the remaining variables are binary (0 or 1), z-score standardization was applied to mitigate any potential biases occurring due to differences in measurement scales. Factor loadings were examined to confirm construct validity, while composite weights for DFL were determined according to the proportion of variance explained by each factor.

**Table 1:** Indicators of digital financial literacy.

Dimensions	Variable	Definitions
**Financial Knowledge (FK)**	FK1	How well do you pay attention to economic and financial information? Highly concerned = 1; Very concerned = 2; Neutral = 3; Rarely concerned = 4; Never concerned = 5
	FK2	Whether to directly answer the interest rate question: Yes = 1; No = 0
	FK3	Is the interest rate question answered correctly? Yes = 1; No = 0.
	FK4	Whether to directly answer the inflation question: Yes = 1; No = 0
	FK5	Is the inflation question answered correctly? Yes = 1; No = 0.
	FK6	Do you understand investment risks? Yes = 1; No = 0
**Financial Skills (FS)**	FS1	Do you have a stock account? Yes = 1; No = 0
	FS2	Do you hold investment funds? Yes = 1; No = 0
	FS3	Do you have a credit card? Yes = 1; No = 0
**Digital Skills (DS)**	DS1	Do you use third-party payment? Yes = 1; No = 0
	DS2	Have you ever purchased internet financial products? Yes = 1; No = 0
	DS3	Have you ever shopped online? Yes = 1; No = 0

As presented in S1 Table, the Kaiser-Meyer-Olkin (KMO) measure of sampling adequacy is 0.810, exceeding the stipulated 0.7 threshold. This implies the dataset is appropriate for factor analysis. Rotated factor loadings for all the variables are listed in S2 Table, while the loadings and variance explained for the three sub-dimensions are outlined in S3 Table.

#### 3.2.3 Control Variables.

Based on the content of the CHFS questionnaire, factors potentially associated with consumption were selected as control variables. Specifically, variables related to household head characteristics (age, education level, urban-rural residence, marital status, and health status), family characteristics (family size, household income, and household assets), and regional characteristics (per capita GDP, government fiscal expenditure, and the share of tertiary industry value-added in GDP).

To absorb local economic conditions and digital infrastructure differences, we construct leave-one-out community averages of household income and years of education (denoted as other_income and other_edu). These variables are calculated as the mean of other households in the same community, excluding the focal household. They serve as proxies for unobserved community characteristics that may correlate with both DFL and consumption.

Table 2 shows the basic statistics of the main variables. CE varies quite a bit across households, with a standard deviation of 1.222. The average DCP and ECP add up to 54.6% (DCP: 0.415, ECP: 0.131). This means non-subsistence spending already accounts for more than half of total household consumption. The DFL index is standardized with a mean of 0 and a standard deviation of 0.584. It ranges from −0.718 to 1.621, indicating that DFL differs widely across households.

**Table 2:** Descriptive statistics.

Variables	Definitions	Min.	Max.	Mean	Std. Dev.
**Dependent variables**					
CE	ln (Total household spending)	2.303	14.059	9.166	1.222
SCP	Proportion of subsistence consumption	0	1	0.455	0.263
DCP	Proportion of developmental consumption	0	1	0.415	0.271
ECP	Proportion of enjoyment consumption	0	1	0.131	0.193
**Independent variables**					
DFL	Digital financial literacy index	−0.718	1.621	0	0.584
Age	age^2^/100	5.290	102.0	40.240	17.049
Education	1 (no school education) −9 (doctoral student)	1	9	3.211	1.603
rural	Rural = 1, Urban = 0	0	1	0.577	0.494
marriage	Married = 1, Others = 0	0	1	0.833	0.373
health	1 (very poor) −5 (excellent)	1	5	3.229	0.995
family_size	total number of family members living together	1	15	3.065	1.526
income	ln (Total household income)	0.560	16.311	10.541	1.408
asset	ln (Total household assets)	0	20.414	12.678	1.762
pergdp	ln (GDP/permanent resident population)	7.987	11.590	10.242	0.804
fiscal	Ln (Fiscal expenditure)	7.271	9.758	8.775	0.511
tertiary	Tertiary industry value-added/GDP	0.465	0.837	0.543	0.079
Other_income	Average income of other households in the same community	4.339	13.348	10.426	0.758
Other_edu	Average education level of other households in the same community	1	9	3.168	0.954
N	14,956				

### 3.3. Model settings

#### 3.3.1 Baseline regression model.

To examine the association between DFL and total consumption, this study uses a linear regression model and employs ordinary least squares (OLS) for parameter estimation. The model specification is as follows:


$$\begin{array}{c} {CE}_{i}=\alpha_{\text{0}}+\alpha_{\text{1}}{DFL}_{i}+\alpha_{\text{2}}{X\text{'}}_{i}+\delta_{i} \end{array}$$ (1)

Where *CE*_*i*_ represents the logarithm of total consumption expenditure. *X’_i_* includes household head characteristics, family characteristics, regional characteristics, and community characteristics. *δ*_*i*_ is the error term.

For the consumption structure, the dependent variables *SCP*_*i*_, *DCP*_*i*_, and *ECP*_*i*_ are shares bounded between 0 and 1. A linear model is not suitable because it may produce predictions outside the unit interval and suffers from heteroskedasticity. Instead, the fractional logit model (FLM) is more appropriate [29], which specifies the conditional expectation as *E*(*y*|*x*) = *G*(*xβ*), where G (·) is the logistic cumulative distribution function (*x* includes the same set of control variables as in equation (1)). The model is estimated by quasi-maximum likelihood with robust standard errors.

#### 3.3.2 Method for exploring potential pathways.

To explore potential pathways through which DFL may be associated with household consumption, we employ the Baron and Kenny (1986) framework to examine whether borrowing, investment, and insurance behaviors are statistically consistent with potential pathways between DFL and household consumption [30]. We then use bootstrap estimation (with 500 replications) to decompose the total association into direct and indirect components and to obtain standard errors for the indirect associations.

In this study, borrowing behavior (*credit*) is measured using a specific variable: whether the family has outstanding current debts (business, education, medical, credit card, or other). A value of 1 is assigned for “yes” and 0 for “no.” Investment behavior (*invest*) is evaluated through the ownership of risk assets. A value of 1 is given if the household head owns such assets—stocks, funds, bonds, derivatives, wealth management products, or non-RMB assets; otherwise, 0 is assigned. Insurance behavior (*insure*) is measured using commercial insurance purchases: a value of 1 is assigned if the household head has bought any commercial insurance products—life insurance, health insurance, or other commercial insurance; otherwise, 0 is assigned.

The analysis is conducted in three steps. First, we estimate the total association between DFL and CE (baseline model). If the coefficient of DFL is statistically significant, the second step examines the association between DFL and each behavioral variable (borrowing, investment, insurance) as specified in equation (2). Third, we estimate the association between each financial behavior and CE, while including DFL as a control variable (Equation 3). The indirect association is computed as the product of the coefficients from the second and third steps. Because of the cross-sectional nature of the data, these estimates are not interpreted as causal mediation but as suggestive evidence of potential pathways. The equations are:


$$\begin{array}{c} M_{i}=\gamma_{\text{0}}+\gamma_{\text{1}}{DFL}_{i}+\gamma_{\text{2}}{X\text{'}}_{i}+e_{i} \end{array}$$ (2)

$$\begin{array}{c} {CE}_{i}=b_{\text{0}}+b_{\text{1}}{DFL}_{i}+b_{\text{2}}M_{i}+b_{\text{3}}{X\text{'}}_{i}+u_{i} \end{array}$$ (3)

Where *M*_*i*_ denotes the behavioral variables (borrowing, investment, or insurance behaviors); *X*_*i*_ includes all control variables; and ei and *u*_*i*_ are error terms.

For consumption structure (proportions bounded in [0, 1]), a linear pathway framework is not appropriate. Following the fractional logit approach used in our baseline specification, examining pathways for consumption structure would require a nonlinear framework.

## 4. Empirical results

### 4.1. Main results

Table 3 presents the baseline regression results for the association between DFL and household consumption.

**Table 3:** DFL and household consumption: Baseline regression.

Variables	OLS		Fractional Logit (AME)					
	Consumption Expenditure		Subsistence Consumption		Developmental Consumption		Enjoyment Consumption	
	(1)	(2)	(3)	(4)	(5)	(6)	(7)	(8)
DFL	1.004***	0.360***	−0.258***	−0.233***	−0.161***	0.010	0.862***	0.454***
	(0.014)	(0.018)	(0.014)	(0.020)	(0.015)	(0.021)	(0.023)	(0.030)
Controls	No	Yes	No	Yes	No	Yes	No	Yes
dy/dx(DFL)			−0.064***	−0.057***	−0.039***	0.002	0.095***	0.048***
			(0.004)	(0.005)	(0.004)	(0.005)	(0.002)	(0.003)
N	14,956	14,903	14,956	14,903	14,956	14,903	14,956	14,903
R-squared	0.230	0.405						

Note: Standard errors in parentheses. *** p < 0.01, ** p < 0.05, * p < 0.1. For fractional logit models, the reported coefficients for DFL are average marginal effects (dy/dx).

Without covariate controls (columns 1, 3, 5, 7), DFL is positively associated with CE and ECP, and negatively associated with SCP and DCP (all p < 0.01). After adding household, regional, and community controls (columns 2, 4, 6, 8), the sign and significance remain stable, except that the DCP coefficient becomes statistically insignificant (column 6). Specifically, a one-unit DFL increase is associated with a 0.360 increase in log CE (p < 0.001), corresponding to roughly 43% higher consumption. For the consumption structure, DFL is related to a reduction in the subsistence share by 5.7 percentage points and an increase in the enjoyment share by 4.8 percentage points, while the association with the developmental share is negligible (0.2 percentage points) and not significant.

Overall, the baseline results are consistent with H1a. Higher DFL is associated with higher CE. For consumption structure, the patterns are clearer for SCP and ECP. The result for DCP is less stable. H1b gets partial support.

### 4.2. Robustness test

To assess the reliability of our baseline associational findings, we conduct a series of robustness checks. Because our baseline results show a significant positive association between DFL and total consumption expenditure (CE), but fragile or insignificant associations with consumption structure variables, our subsequent checks focus primarily on the CE estimates.

Specifically, we first implement propensity score matching (PSM) to address selection on observables, construct an alternative DFL measure to mitigate measurement bias, and impose sample restrictions to exclude households employed in the digital or financial sectors. We also re-estimate the baseline model after removing medical expenditures from CE. Finally, as a supplementary check for potential endogeneity concerns, we employ an Instrumental Variable (IV) approach, complemented by Conley and Oster analyses. A separate sensitivity check regarding the definition of the consumption structure (excluding medical expenditures from the developmental category) is provided at the end of this section (see S4 Table).

#### Propensity score matching.

To address selection on observables, we use nearest-neighbor, radius, and kernel matching to estimate the average treatment effect on the treated (ATT). All three methods give positive and significant ATT estimates at the 1% level (Table 4). Even after balancing covariates such as age, education, and income, the treated group consistently shows higher expenditure than the matched controls. This is consistent with our main regression results, mitigating concerns about selection bias.

**Table 4:** Average Treatment Effect on the Treated (ATT) for CE.

Matching method	Treated	Controls	ATT	S.E.	T-stat
1:4 Nearest Neighbor	9.697	9.402	0.296	0.036	8.120***
Radius (caliper = 0.01)	9.697	9.369	0.305	0.030	10.040***
Kernel	9.675	9.371	0.304	0.031	9.970***

Notes: *** p < 0.01. Standard errors are bootstrapped (500 replications). The treated group is defined as households with DFL above the median (high DFL).

#### Alternative DFL measure.

To mitigate potential measurement bias arising from the weighting scheme, we construct a simplified DFL index (DFL_new) by summing the scores of the 12 items directly. Table 5, column (1), shows that DFL_new retains a positive and significant association with CE, consistent with our baseline findings.

**Table 5:** Robustness test results.

Variables	(1)	(2)	(3)
	Alternative Explanatory Variable	Excluding special occupations	Excluding Medical Expenditures
DFL_new	0.151***		
	(0.007)		
DFL		0.372***	
		(0.018)	
DFL			0.401***
			(0.019)
Controls	Yes	Yes	Yes
R-squared	0.407	0.405	0.474
N	14,903	14,745	14,903

Note: Standard errors in parentheses.* p < 0.1, ** p < 0.05, *** p < 0.01.

#### Sample restriction.

Households with members working in the digital or financial sectors (e.g., IT, banking) may possess unobserved advantages. After excluding these households, Column (2) shows that the coefficient of DFL remains largely unchanged, indicating that our results are not attributable to occupational bias.

#### Excluding medical expenditures.

Medical expenditures are often involuntary (e.g., due to illness) rather than a discretionary choice reflecting financial literacy. We re-estimate the model after subtracting all medical expenditures from CE. As shown in Column (3), the DFL coefficient remains positive and significant, indicating that the association holds even for discretionary consumption.

As a further robustness check, we also examine whether the classification of consumption structure relates to the null results. Specifically, we recalculate the consumption structure proportions excluding medical expenditures from the developmental category. The results are reported in S4 Table. After removal, the association between DFL and DCP becomes positive and significant (AME = 0.025, p < 0.01), whereas the baseline estimate was insignificant (AME = 0.002). SCP and ECP remain qualitatively unchanged. This indicates that the null result for the original developmental category is sensitive to the inclusion of healthcare expenditure, which is largely involuntary and less associated with DFL. Therefore, we interpret the result for DCP with caution: DFL may be positively associated with the proportion of voluntary developmental expenditure (education, transport, communication), even though the association with the aggregate category (including healthcare) is not robust.

#### Instrumental variable estimation.

To address potential endogeneity of DFL, we use the average DFL of other household heads in the same community (OtherDFL), constructed using the leave-one-out method, as an instrumental variable (IV). Theoretically, the DFL level of other household heads is strongly correlated with the respondent’s own DFL, because peer interactions and social learning through information transmission are associated with higher individual DFL [31]. Regarding exogeneity, community-average DFL may be related to individual consumption through two channels: (i) an indirect channel, namely peer interactions that are associated with higher individual DFL, and (ii) direct channels such as community public services, digital infrastructure, or consumption norms. Existing research shows that peer effects in financial decisions operate largely through information transmission [30], which is consistent with the indirect channel. However, we cannot rule out direct channels entirely.

To reduce this concern, we use community average income and education (constructed via leave-one-out) as comprehensive proxies for unobserved digital infrastructure and financial access, which are not directly available in our CHFS dataset. In China, broadband penetration and bank branch density are systematically correlated with local income levels and educational attainment [32–34]. As these two variables are already included as controls in our baseline specification, they also enter the first and second stages of the IV estimation, thereby absorbing much of the regional variation that might otherwise violate the exclusion restriction.

We use Two-Stage Least Squares (2SLS) for the continuous outcome (Consumption Expenditure) and Two-Stage Residual Inclusion (2SRI) for the fractional outcomes (Consumption Structure). The first stage regresses DFL on the instrument (OtherDFL) and all controls to obtain the residuals (v_hat). For 2SRI, these residuals are then included as an additional regressor in the second-stage fractional logit model to correct for endogeneity bias (for 2SLS, the standard procedure uses the predicted DFL). As anticipated, the first-stage regression in Table 6, Column 1, suggests a strong positive association between OtherDFL and individual DFL. The instrument is not weak, satisfying the first-stage relevance requirement for IV estimation.

**Table 6:** Instrumental variable estimates of DFL on household consumption.

Variables	(1)	(2)	(3)	(4)	(5)
	DFL (First Stage)	Consumption Expenditure (2SLS)	Subsistence Consumption (2SRI)	Developmental Consumption (2SRI)	Enjoyment Consumption (2SRI)
OtherDFL	0.359***				
	(0.022)				
DFL		0.705***	−0.338**	−0.202	1.044***
		(0.138)	(0.160)	(0.165)	(0.236)
v_hat			0.107	0.217	−0.599***
			(0.162)	(0.168)	(0.238)
Controls	Yes	Yes	Yes	Yes	Yes
dy/dx(DFL)			−0.082 **	−0.048	0.111**
			(0.039)	(0.039)	(0.025)
R-squared	0.478	0.391			
Wald χ²			941.95	1400.18	3096.02
F value	263.25				
Kleibergen‑Paap rk LM	98.45 [p=0.000]				
Durbin-Wu‑Hausman endogeneity test	χ²(1)=8.76 [p=0.003]				
N	14,903	14,903	14,903	14,903	14,903
Conley UCI (γ ≤ 0.1)		[0.198, 1.220]	[-0.426, 0.269]	[-0.389, 0.307]	[-0.212, 0.452]
Oster δ (rmax = 1)		0.152	0.051	−0.003	0.083

Note: Standard errors in parentheses.* p < 0.1, ** p < 0.05, *** p < 0.01.

In the second stage, the 2SLS estimate for total consumption indicates that a one-unit increase in DFL is associated with a 0.705 increase in log total consumption expenditure (cluster‑robust SE = 0.138, z = 5.12, p < 0.001), more than double the OLS estimate of 0.360 (SE = 0.018). The Durbin-Wu‑Hausman test rejects exogeneity of DFL (χ²(1) = 8.76, p = 0.003), suggesting OLS is inconsistent. These IV estimates provide supporting evidence that the positive association between DFL and total consumption is robust to potential endogeneity concerns.

For the consumption structure, the results are less clear. Column (3) shows that for SCP, the AME of DFL is −0.082 (p = 0.034). For DCP (column 4), the AME is −0.048 (p = 0.221). For ECP (column 5), the AME is 0.111 (p < 0.001). Thus, the IV results show a robust positive association of DFL with CE, whereas the effects on associations with consumption structure, though consistent with expectations, are not robust enough.

To further assess the robustness of the IV estimates, we conduct two additional sensitivity analyses. We acknowledge that direct measures of community-level GDP per capita, broadband penetration, or financial outlet density would be preferable, but such data are not available in the CHFS. Therefore, we complement the proxy approach using community average income and education (described above) with a formal sensitivity analysis following Conley et al. [35]. Regarding the likely direction of bias, if communities with higher average DFL also have better digital infrastructure or stronger consumption norms, the OLS estimate of DFL on consumption would be upward biased because it captures both the individual DFL effect and the positive community effect [36]. Hsu et al. (2012) caution that using area-level averages as proxies for individual characteristics may be correlated with the instrument, affecting 2SLS bias [37]. Consequently, the IV estimate under the exclusion restriction could be biased in either direction, depending on the correlation between the instrument and the omitted direct channel.

To provide a quantitative benchmark for the Conley sensitivity analysis, we first estimate the reduced-form relationship between the instrument (OtherDFL) and log total consumption expenditure, conditional on all controls. The reduced-form coefficient is 0.253 (p < 0.001). This implies that the chosen bound |γ| ≤ 0.1 allows the instrument to have a direct effect on consumption equivalent to approximately 40% of the reduced-form total effect (0.1 / 0.253). We consider this a generous and conservative allowance, as it substantially exceeds what one might reasonably attribute to indirect community-level channels such as local consumption norms or digital infrastructure spillovers.

To examine how sensitive our IV estimates are to modest departures from the exclusion restriction, we allow the instrument to have a direct effect on consumption (e.g., through unobserved local conditions) up to a magnitude of |γ| ≤ 0.1. We then compute the Union Confidence Intervals (UCI) for the DFL coefficient. As shown in Table 6, the UCI for CE (2SLS) is [0.198, 1.220], which lies entirely above zero. This suggests that the positive association between DFL and total consumption holds even under modest violations of the exclusion restriction. In contrast, the UCIs for the consumption structure variables (SCP, DCP, and ECP) all include zero, indicating that those structural estimates are fragile.

For the OLS specifications reported in Table 3, we apply the Oster (2019) procedure as a supplementary check for omitted variable bias [38]. The Oster δ indicates how much stronger unobserved selection would need to be (relative to observed controls) to drive the OLS coefficient to zero. For CE, the coefficient drops from 1.004 to 0.360 after including controls (Table 3, columns 1–2), yielding a δ of 0.152. While this value implies that the result is not immune to selection bias, it is notably larger than those for SCP (0.051) and ECP (0.083) (See Table 6). For DCP, δ is negative (−0.003), pointing to an unstable specification. For consumption structure, we apply the Oster test within a linear probability model as a conservative reference.

To sum up, the robustness checks indicate that the positive link between DFL and total consumption expenditure is stable in both sign and significance, appearing reasonably robust to potential endogeneity concerns. Thus, Hypothesis 1a is strongly supported. In contrast, the estimates for the consumption structure are not robust; therefore, Hypothesis 1b is not supported for the originally defined consumption structure.

### 4.3. Exploring potential pathways

As theorized above, DFL may be associated with household consumption through borrowing, investment, and insurance. Following the pathway approach (equations 2 and 3), we limit the pathway analysis to total consumption expenditure because DFL’s associations with consumption structure are not robust. Hence, we do not test the consumption‑structure part of H2a–H2c. Table 7 reports the pathway analysis results for total consumption expenditure.

**Table 7:** Pathway analysis results for CE.

Variables	(1)	(2)	(3)	(4)	(5)	(6)	(7)
	CE	Borrowing (logit)	CE	Investment (logit)	CE	Insurance (logit)	CE
DFL	0.373***	0.955***	0.337***	2.714***	0.357***	0.809***	0.361***
	(0.018)	(0.048)	(0.018)	(0.088)	(0.019)	(0.070)	(0.018)
Credit			0.233***				
			(0.020)				
Invest					0.068***		
					(0.025)		
Insure							0.165***
							(0.026)
Controls	Yes	Yes	Yes	Yes	Yes	Yes	Yes
R-squared	0.407		0.408		0.403		0.404
dy/dx(M)		0.139***		0.186***			0.060***
		(0.006)		(0.005)			(0.005)
N	14,903						

Note: Standard errors in parentheses.* p < 0.1, ** p < 0.05, *** p < 0.01.

As shown in Table 7, borrowing has the largest indirect association (≈0.032), followed by investment (≈0.013) and insurance (≈0.010). These values are computed as the product of the AME of DFL on each financial behavior and that behavior’s coefficient in the CE regression. All three are statistically significant (p < 0.001 for borrowing and insurance, p = 0.004 for investment), and their bootstrap confidence intervals exclude zero. These patterns are consistent with the theoretical framework and partially support H2a, H2b, and H2c for total consumption expenditure.

### 4.4. Heterogeneity analysis

Given the robust positive association between DFL and CE, we examine whether this relationship varies across different subgroups. We conduct heterogeneity analyses by urban-rural residence, region (Eastern, Central, Western, and Northeastern China), household income level, and educational attainment.

In 2019, per capita consumption in rural China (CNY 13,328) was much lower than in urban areas (CNY 28,063), reflecting structural imbalances. So, we run separate analyses for rural and urban subsamples. Regional economic development varies greatly across China, which is associated with infrastructure and digital finance. To test regional differences, we follow the CHFS classification and divide the sample into Central, Eastern, Western, and Northeastern areas, with Central as the base group. Income is a key factor associated with consumption according to liquidity constraint theory and the life‑cycle hypothesis. We group households into low, middle, and high income categories using the 25th and 75th percentiles of total household income (CNY 18,312 and CNY 95,429, respectively). Finally, education matters because DFL itself combines digital skills and financial literacy, and education plays a role in both [18]. Education is related to learning capacity and reasoning. We divide the sample into low (junior high school or below), medium (senior high school to junior college), and high (undergraduate or above) education groups.

Table 8 presents the results of the heterogeneity analyses, reporting the average marginal effects (AME) of DFL on CE across subgroups. (Full regression results, including interaction terms, are available upon request.)

**Table 8:** Heterogeneity analysis results.

Subgroup	Categories	Marginal Effect (dy/dx)	95% CI	Interaction coefficient	p-value
Urban-Rural	Urban	0.352*** (0.019)	[0.315, 0.389]	Reference	—
	Rural	0.383*** (0.021)	[0.342, 0.425]	0.056	0.076
Region	Central	0.336*** (0.032)	[0.273, 0.399]	Reference	—
	Eastern	0.371*** (0.024)	[0.324, 0.418]	0.036	0.326
	Western	0.371*** (0.027)	[0.317, 0.425]	0.035	0.356
	Northeastern	0.330*** (0.040)	[0.251, 0.409]	−0.005	0.913
Income	Low	0.481*** (0.043)	[0.399, 0.563]	Reference	—
	Middle	0.300*** (0.022)	[0.257, 0.343]	−0.182	<0.001
	High	0.407*** (0.029)	[0.350, 0.464]	−0.075	0.127
Education	Low	0.390*** (0.022)	[0.347, 0.433]	Reference	—
	Medium	0.348*** (0.026)	[0.297, 0.399]	−0.041	0.200
	High	0.395*** (0.070)	[0.258, 0.532]	0.006	0.934

Notes: *** p < 0.01, ** p < 0.05, * p < 0.1. The interaction p-value is for the test of difference from the reference category. Standard errors in parentheses. All models include full controls.

We first examine whether the relationship between DFL and CE differs between urban and rural areas. The AME for urban households is 0.352 (p < 0.001); for rural households, it is 0.383 (p < 0.001). The interaction term (DFL × rural) is positive and marginally significant at the 10% level (coefficient = 0.056, p = 0.076), suggesting a potential but weak difference between urban and rural areas.

Turning to regional differences, the AMEs are 0.336 (Central), 0.371 (Eastern), 0.371 (Western), and 0.330 (Northeast), all p < 0.01. All interaction terms are insignificant (p > 0.1), indicating that the relationship between DFL and CE does not vary significantly across regions.

For income level, the AME is 0.481 (low income), 0.300 (middle income), and 0.407 (high income), all p < 0.01. The middle-income interaction term is negative and significant (coefficient = –0.182, p < 0.001), indicating a significantly weaker association than for low-income households. The difference between low-income and high-income is not significant (p = 0.127). Accordingly, the association is strongest for low-income households, followed by high-income, while middle-income households show the weakest relationship.

Regarding educational attainment, the AMEs are 0.389 (low), 0.348 (medium), and 0.395 (high), all p < 0.01. None of the interaction terms are significant (p > 0.1), implying that the association does not vary by education level.

In summary, DFL exhibits a robust positive association with CE across all examined subgroups. The association is most pronounced for low-income households. In contrast, regional and educational differences are not significant, and the urban–rural gap is only marginally significant.

## 5. Discussion and conclusion

### 5.1. Discussion

First, DFL is positively associated with CE, and this association is robust. DFL is associated with households relaxing liquidity constraints via borrowing, generating property income through investment, and reducing precautionary savings through insurance—three channels that are jointly associated with higher disposable resources for current consumption. This result is consistent with recent CHFS-based studies showing that digital financial capability is associated with higher total household consumption and online spending, and is partly related to eased credit constraints [22], and that digital finance is related to recurring expenditures [39]. The present study extends this literature by demonstrating that the positive association survives a range of stringent identification strategies, including instrumental variable estimation and sensitivity checks (Conley UCI, Oster).

Second, the positive link is strongest for low-income households. This pattern is consistent with the fact that disadvantaged groups typically face greater barriers to accessing formal financial services; consequently, improvements in digital financial skills relate to higher marginal returns for these populations. The inclusive nature of digital finance is well documented: existing studies indicate that digital tools are associated with financially underserved households overcoming liquidity constraints and limited access to formal services [23,40]. Furthermore, our findings are consistent with the Technology Acceptance Model and Perceived Risk Theory [41,42]. For low-income individuals, enhanced DFL is related to higher perceived ease of use and perceived usefulness of digital financial tools and lower perceived risks, which in turn correlate with greater willingness to use digital channels for consumption. Collectively, these patterns are consistent with low-income households showing the strongest links between DFL and consumption.

Third, among the three financial behaviors we examine, borrowing is the main pathway between DFL and CE, while investment and insurance play smaller roles. This finding can be explained by the differing time horizons of the three behaviors. Borrowing provides immediate liquidity, whereas investment returns typically materialize over a longer period, and insurance is associated with reduced precautionary savings rather than directly funding current consumption. This pattern is consistent with liquidity constraint theory [2]. In practice, DFL is correlated with more accurate evaluation of credit terms, avoidance of unfavorable loan conditions, and more effective access to digital credit products [17,18]. Our analysis echoes Meng (2024), who identifies the easing of credit constraints as a key channel between digital financial capability and household consumption. The smaller indirect associations through investment and insurance are also consistent with theoretical expectations: property income accumulates gradually, and insurance liberates resources only indirectly. At a broader theoretical level, human capital theory offers a useful framework: DFL can be viewed as a specialized form of human capital [43] that is related to financial comprehension and technical proficiency, which in turn are linked to more effective borrowing and investing.

Fourth, this study is the first, to our knowledge, to distinguish between total consumption expenditure and consumption structure in the DFL literature. We find that DFL’s associations with consumption structure are not robust. These associations become statistically insignificant when key identification assumptions are relaxed. This result stands in contrast to some earlier studies that reported significant structural shifts [14,37]. The discrepancy likely arises for two reasons: first, the present study applies stricter sensitivity checks (Conley UCI and Oster), which reveal that structural estimates are fragile when faced with violations of the exclusion restriction or omitted variable bias; second, structural shifts in consumption patterns may require longer time horizons than cross-sectional data can capture. Focusing on the developmental category, a sensitivity check that excludes medical expenditures makes the DFL–developmental consumption association positive and significant, indicating that the baseline null result is driven by largely involuntary medical expenditure. Hence, DFL may be positively associated with more discretionary developmental spending (education, transport, communication) but not with the aggregate category including healthcare. Given the fragility of these estimates, we caution against drawing strong causal inferences about consumption structure using cross-sectional data.

### 5.2. Conclusion

Using the 2019 China Household Finance Survey and a battery of identification strategies (instrumental variables, sensitivity analyses, mediation tests, and propensity score matching), we provide the following evidence-based answers to three interrelated questions: (i) whether digital financial literacy is associated with household total consumption expenditure and consumption structure, (ii) through which financial behaviors (borrowing, investment, insurance) these associations operate, and (iii) whether the association varies across household subgroups.

First, DFL is positively correlated with total consumption expenditure, and this relationship is supported by stringent endogeneity checks (Conley UCI, Oster) and robustness tests. Second, DFL relates to a lower SCP and a higher ECP, yet the evidence for a systematic shift in the full consumption structure (including DCP) is not robust. Third, borrowing is the main pathway between DFL and total consumption, while investment and insurance play much smaller roles. Fourth, the association is strongest for low-income households, with only marginal urban–rural differences and no significant variation by region or education.

These findings also carry clear policy implications. Given that DFL is robustly associated with higher total consumption, especially among disadvantaged groups, governments should integrate digital financial literacy programmes into broader demand-side policies. Given that borrowing is the primary channel, it is essential to facilitate responsible digital credit by ensuring transparent terms, providing borrower education, and strengthening consumer protection. The non-robust findings for the consumption structure imply that DFL alone will not automatically shift spending toward enjoyment or developmental goods; complementary investments in education, health, and cultural services remain necessary. Finally, digital insurance alone appears to have limited associations with consumption; building trust and risk awareness may be important for insurance to be associated with reduced precautionary savings.

In sum, this study contributes to the debate on digital finance and consumption by showing that DFL is positively associated with total spending and negatively associated with inequality, but not with structural changes in consumption. Enhancing DFL, especially among low-income households, and coupling it with responsible credit mechanisms offers a promising pathway toward more inclusive and sustainable household consumption growth.

## 6. Limitations and future directions

Despite providing valuable empirical analysis of the relationship between digital financial literacy and household consumption, this study has several limitations.

First, the cross-sectional design (2019 CHFS) positions our findings as associative evidence. Reverse causality cannot be fully ruled out even with IV estimation. Moreover, the consumption structure and pathway analyses are exploratory: the estimates are not robust to model specifications or identification assumptions, and the Baron–Kenny decomposition is descriptive rather than indicating causal pathways. The exclusion restriction of the instrumental variable is inherently untestable; our defense therefore relies on conceptual arguments, proxy controls, and sensitivity analysis (Conley UCI). Future research with panel data or stronger identification strategies, such as instrumental variables for each behavioral variable or quasi-experimental approaches, would help provide stronger evidence for the associations examined in this study.

Second, measurement issues exist. Financial behaviors are captured as binary variables, ignoring intensity or frequency; more detailed measures (e.g., amounts, transaction counts) are desirable. Additionally, DFL relies on self-reported answers, which may introduce subjective bias; combining survey data with objective digital footprints or administrative records could improve measurement. Furthermore, our DFL index does not directly measure digital security behaviors (e.g., antivirus use, phishing avoidance) or algorithmic literacy (e.g., understanding robo-advisor recommendations), as the 2019 CHFS data did not include such items. Consequently, our measure captures what Lyons and Kass-Hanna (2021) term ‘basic digital financial literacy’ rather than the full spectrum of advanced competencies. Future research using more recent survey waves should refine the measurement of both DFL and financial behaviors.

Third, this study focuses solely on China, which limits generalizability. Future research could adopt cross-cultural or cross-national comparative approaches to explore how institutional contexts, digital infrastructure, and cultural norms shape the DFL‑consumption nexus.

## Supporting information

S1 Table KMO and Bartlett Tests. (DOCX)

S2 Table Rotated factor loadings and unique variances. (DOCX)

S3 Table Variance explained for sub-dimensions. (DOCX)

S4 Table Consumption structure estimates: baseline vs. excluding medical expenditures. (DOCX)

## Abbreviations

AME: Average marginal effect
CE: Total consumption expenditure
ECP: Enjoyment consumption proportion
DCP: Developmental consumption proportion
DFL: Digital financial literacy
SCP: Subsistence consumption proportion
