# Supplementary material for: Digital financial literacy and household consumption: Total expenditure versus consumption structure in China
Source: PLoS One. 2026 Sep 1;21(9):e0357484. doi: 10.1371/journal.pone.0357484 (PMC13533390; doi:10.1371/journal.pone.0357484)
Supplement: Supplementary file 1 [file pone.0357484.s001.docx]

**S1 Table. KMO and Bartlett Tests.**

| **Number of KMO sampling** | | 0.810 |
| --- | --- | --- |
| **Bartlett's sphericity test** | **Approximate chi square** | 47168.133 |
|  | **Free degree** | 66 |
|  | **P value** | 0.000 |
