# Supplementary material for: Digital financial literacy and household consumption: Total expenditure versus consumption structure in China
Source: PLoS One. 2026 Sep 1;21(9):e0357484. doi: 10.1371/journal.pone.0357484 (PMC13533390; doi:10.1371/journal.pone.0357484)
Supplement: Supplementary file 2 [file pone.0357484.s002.docx]

**S2 Table. Rotated factor loadings and unique variances.**

| **Variables** | **Factor1** | **Factor2** | **Factor3** | **Uniqueness** |
| --- | --- | --- | --- | --- |
| FK1 | 0.269 | 0.202 | 0.270 | 0.814 |
| FK2 | 0.733 | 0.231 | 0.064 | 0.406 |
| FK3 | 0.541 | 0.161 | 0.101 | 0.671 |
| FK4 | 0.800 | 0.139 | 0.040 | 0.339 |
| FK5 | 0.711 | 0.182 | 0.126 | 0.446 |
| FK6 | 0.274 | 0.170 | 0.044 | 0.894 |
| FS1 | 0.185 | 0.171 | 0.527 | 0.658 |
| FS2 | 0.098 | 0.087 | 0.375 | 0.842 |
| FS3 | 0.153 | 0.426 | 0.292 | 0.710 |
| DS1 | 0.196 | 0.757 | 0.041 | 0.387 |
| DS2 | 0.140 | 0.365 | 0.248 | 0.785 |
| DS3 | 0.220 | 0.770 | 0.087 | 0.352 |
