# Supplementary material for: Digital financial literacy and household consumption: Total expenditure versus consumption structure in China
Source: PLoS One. 2026 Sep 1;21(9):e0357484. doi: 10.1371/journal.pone.0357484 (PMC13533390; doi:10.1371/journal.pone.0357484)
Supplement: Supplementary file 3 [file pone.0357484.s003.docx]

**S3 Table. Variance explained for sub-dimensions.**

| **Factors** | **Sub-Dimension** | **Kernel Variable (loading>0.50)** | **Variance Explained** | **Cumulative Interpretation Rate** |
| --- | --- | --- | --- | --- |
| f1 | Financial Awareness | FK2, FK4, FK5 | 48.89% | 48.89% |
| f2 | Usage of Digital Tools | DS1, DS3 | 36.58% | 85.47% |
| f3 | Usage of Traditional Financial Instruments | FS1 | 14.53% | 100.00% |
