# Supplementary material for: Digital financial literacy and household consumption: Total expenditure versus consumption structure in China
Source: PLoS One. 2026 Sep 1;21(9):e0357484. doi: 10.1371/journal.pone.0357484 (PMC13533390; doi:10.1371/journal.pone.0357484)
Supplement: Supplementary file 4 [file pone.0357484.s004.docx]

**S4 Table. Consumption Structure Estimates: Baseline vs. Excluding Medical Expenditures.**

| **Consumption Structure** | **Baseline AME** | **Excluding medical expenditures AME** | **Difference (Excluding- Baseline)** |
| --- | --- | --- | --- |
| Subsistence Consumption | -0.058*** | -0.078*** | -0.020 |
|  | (0.004) | (0.005) |  |
| Developmental Consumption | 0.002 | 0.025*** | 0.023 |
|  | (0.005) | (0.004) |  |
| Enjoyment Consumption | 0.049*** | 0.053*** | 0.004 |
|  | (0.003) | (0.003) |  |

Notes: Standard errors in parentheses. *** p <0.01, ** p <0.05, * p <0.1. The developmental consumption in the baseline model includes medical expenditures; after exclusion, it includes only education, transport, and communication.
